# Knowledge, uptake of preconception care and associated factors among reproductive age group women in West Shewa zone, Ethiopia, 2018

**DOI:** 10.1186/s12905-020-00900-2

**Published:** 2020-02-19

**Authors:** Daniel Belema Fekene, Benyam Seifu Woldeyes, Maru Mossisa Erena, Getu Alemu Demisse

**Affiliations:** 1 Department of Midwifery, College of Medicine and Health Sciences, Ambo University, Ambo, Ethiopia; 2 Department of Public health, College of Medicine and Health Sciences, Ambo University, Ambo, Ethiopia

**Keywords:** Preconception care, Knowledge, Uptake, Reproductive age, West shewa, Ethiopia

## Abstract

**Background:**

Preconception care is a set of interventions that are to be provided before pregnancy, to promrote the health and well-being of womwen and couples .

**Methods:**

A community-based cross-sectional study was employed among a sample of 680 reproductive-aged women in the West Shewa zone, Oromia regional state, from November 2017 until the end of January 2018. The data were collected using a pre-tested and structured questionnaire. The collected data were coded and entered into Epi data version 3.1 and exported to SPSS 22 for analysis. Bivariate and multivariate logistic regression models were utilized to determine factors associated with the outcome variable. The association was presented in odds ratio with 95% confidence interval and significance determined at a *P*-value less than 0.05.

**Result:**

A total of 669 participants had participated with a response rate of 98.3%. Among the respondants, only 179 (26.8%) had a good knowledge of PCC and 97 (14.5%) of them had utilized PCC. Factors that show significant association with good knowledge of PCC are history of institutional delivery (AOR = 1.43 (95%CI (1.31–7.33), PNC service utilization, (AOR = 5.02 (95%CI (3.22–7.84), history of using modern contraceptive, (AOR = 1.44 (95%CI (1.37–6.98) higher educational status (AOR = 4.12 (95%CI (1.22–6.52) and being regularly employed (AOR = 1.8 (95%CI (1.01–3.22). Factors like better family monthly income (AOR = 4.1 (95%CI (1.57–9.35), history of PNC (AOR = 6.33 (95%CI (3.94–10.17) and good knowledge of PCC (AOR = 4.3 (95%CI (2.67–6.98) had shown positive association towards the uptake of PCC.

**Conclusion and recommendation:**

This study found that only one-quarter of the women in the study has good knowledge of PCC and low uptake of PCC. History of institutional delivery, PNC service utilization, history of using a modern contraceptive, educational status and being regularly employed were factors that affect knowledge of PCC and family income, having good knowledge about PCC and history of PNC were affect the uptake of PCC.Therefore, PCC needs serious attention from the government and other stakeholders.

## Background

Preconception care (PCC) is a set of interventions that are to be provided before pregnancy, to promote the health and well-being of women and couples. It is aimed at taking care of women and couples before conception occurs. Integrating PCC components into routine primary care visits can improve maternal and child health, in both the short and long term [1, 2].

According to world health organization (WHO), the recommended areas to be addressed by the PCC package are nutritional conditions (Screening for anemia and diabetes, Supplementing iron and folic acid, Information, education, and counseling and Monitoring nutritional status), Tobacco use, genetic condition, environmental health, infertility/ subfertility, interpersonal violence, too-early, unwanted and rapid successive pregnancies, Sexually transmitted infections (STIs), HIV, and mental health [2].

About 830 women die from pregnancy and childbirth-related complications around the world every day. In 2015 women 303, 000 died from pregnancy and childbirth-related problems [3]. Most of these complications develop during pregnancy, exist before and worsened during pregnancy, especially if not managed as part of the PCC [4]. Ethiopia Health policies, strategies, and programs are preventive rather than curative. And it aimed at addressing the anticipated and present health issues and problems in the country [5]. But in Ethiopia, the pregnancy-related mortality ratio was 412 per 100,000 live births and the lifetime risk of pregnancy-related death is 21 in 1000 women [6]. According to Ethiopian demographic, health survey (EDHS) 2016, 22% of women age 15–49 are thin (with a BMI less than 18.5), while 8% are overweight or obese. More than half of children age 6–59 months (57%) and 24% of women age 15–49 are anemic [6]. This risk of maternal and infant mortality and pregnancy-related complications can be reduced by increasing access to quality preconception and inter conception care like skilled birth attendants [7].PCC is one of the proven strategies on the reduction in mortality and decreases the risk of adverse health effects for the woman, fetus, and neonate by optimizing maternal health services and improves woman’s health [8]. Knowledge and uptake of PCC can be obtained from experience, health care providers, family, relatives, and media. Studies revealed that women who received PCC have more knowledge, uptake PCC service and often show risk alleviation behaviors [9]. Identifying the knowledge and uptake of the PCC at the local context is a very crucial and timely issue, this can accelerate the reduction in maternal and neonatal mortality for progress towards sustainable development goals (SDGs). The study conducted in Ethiopia regarding the knowledge and utilization of PCC was limited to urban towns [10, 11], while preconception in the districts (urban and rural) gained little attention. Considering the scarcity of reliable and documented evidence on the knowledge and uptake of PCC in the study area, this study aims to identify the level of women’s knowledge, uptake and associated factors of PCC. This will help in estimating the PCC needs of reproductive age group women and their uptake of preconception service.

## Methods

### Study area and period

The study was conducted in the West Shewa zone of Oromia regional state, Ethiopia, from November 2017 to the end of January 2018. West Shewa zone has 24 woredas and the woredas are sub-classified into urban and rural kebeles (the smallest administrative unit). According to the information obtained from the zonal health office in 2017/2018, the West Shewa zone has a total population of 2,058,676, of whom 1,028,501 are men and 1,030,175 women. Out of this, the total women in the reproductive age group were 447,042. All reproductive age women in a West Shewa zone where source population and all reproductive age group women who are married, living in union, fecund pregnant women and who lives in the zones for more than 6 months were included.

### Sample size and sampling procedures

The sample size was calculated with Epinfo version 7.1 stat calc for a cross-sectional study design using the assumption [Zα/2 = 1.96, a margin of error 5% *P* = 28%; Women’s knowledge and associated factors in PCC [8], design effect of 2]. By adding a 10% non-response rate, the final sample size becomes 680. A multistage, stratified sampling procedure was employed. In the first stage, 8 woredas from the 24woredas in the zone were selected using a lottery method. In the second stage, one urban and one rural kebeles from each woreda were randomly selected. In the third stage, from those selected kebeles, households which reproductive-age women were live in were selected randomly from the sampling frame obtained from kebele health office and health extension workers. The sample size for each kebeles was determined proportionally to the number of women’s reproductive age groups within each kebeles. In the case of more than one eligible woman were encountered in the selected household, a lottery method was used to determine which woman would be interviewed.

### Data collection tool, quality control and measurement

A structured, interview administered questionnaire was used to collect data. The questionnaire was prepared in English(Additional file 1) and translated into local language, Afan Oromo by the translator, and then translated back to English by a third person to check for consistency. The tool adapted from previous literature in different parts of the world and modified according to the local context [10–14]. Eight nurses were recruited as data collectors and Assistant professors with a background of health professionals were hired as supervisors. In addition, the data collectors were trained for 1 day on the techniques of data collection and the purpose of the study for study participants before the start of data collection. Pre-test was done on 5% of the total study participant and necessary adjustment was made. The data was collected house to house using an interview questionare. Data completeness and consistency were checked, cleaned and compiled by the supervisors on a daily basis. Incomplete data were removed from the study.

### Measurements

The knowledge level of the study participants was determined using a dichotomous scale. Eleven knowledge related items were used to assess women’s knowledge on PCC and the question was scored out of twenty points. With a 50% cut of point women’s knowledge was divided into two. Those participants who have scored 10–20 of correct responses to PCC knowledge questions were considered as having good knowledge while those who scored less than 10 of correct responses considered poor knowledge [10, 12].

The uptake of PCC was determined, if the women received PCC at least once types of intervention either advice or treatment, and lifestyle modification care (screened for any disease and get treatment, take folic acid, take the vaccine, get counseling, modify diet, cessation of alcohol, cessation of cigarette smoking, stop taking illegal drugs, free from, create healthy environment) before being pregnant [10, 13].

#### Data management and analysis

Data were entered into Epi-Data Version 3.1 and exported to SPSS version 22 for analysis. Factors were tested using the bivariable analysis, and *p*-value≤0.2 was a candidate for the multivariable logistic regression analysis. To descriptive statistics; frequencies and percentages were used. Binary logistic regression analysis to examine the crude association of predictors on the desire to use PCC and knowledge about PCC, then multiple logistic regressions to see the effect of predictors on the desire to use PCC and knowledge about PCC and Odds ratio, 95% CI and *P*-value 0.05 were used.

## Result

### Socio-demographic characteristic

A total of 669 participants had fully responded to the questionnaire making a response rate of 98.3%. The mean age of the respondents was 25.59 with the standard deviation of ±2. 89 years. The study participants were predominantly Oromo 547 (81.8%) and protestant 353 (52.8) by their ethnicity and religion respectively. From the total respondents, the majority of them were married 572 (85.5%) and 249 (37.2%) of them were housewives. 272 (40.7%) were getting a monthly income of less than one thousand five hundred Ethiopian Birr.(Table 1).

**Table 1:** Socio-demographic characteristics of reproductive age group of women in selected woreda of WestShewazone,Oromia, regional state, 2018

Variables	Frequency	Percentage
Age categories		
18–22	98	14.7
23–27	128	19.1
28–32	116	17.3
33–37	174	26.0
38–42	110	16.4
43–49	43	6.4
Religion		
Orthodox	250	37.4
Protestant	353	52.8
Muslim	58	8.7
Catholic	4	0.6
Other	4	0.6
Ethnicity		
Oromo	547	81.8
Amhara	90	13.5
Gurage	6	0.9
Tigre	26	3.9
Occupation		
Housewife	249	37.2
Student	26	3.9
Government employee	135	20.2
NGO employee	92	13.8
Private business	167	25
Marital status of the women		
Married	572	85.5
Divorced	64	9.6
Widowed	17	2.5
Cohabited	16	2.4
Educational status		
No formal school	84	12.6
1–4 grade completed	92	13.8
5–8 grade completed	208	31.1
9–12 completed	169	25.3
College & above	116	17.3
Monthly income		
< = 1500	272	40.7
1501–2000	182	27.2
2001–2800	51	7.6
2801+	164	24.5

### Past obstetrics characteristics

In this study, 479 (71.6%) of the participants had at least one pregnancy before. The majority 349 (72.8%) of participants had visited health facilities for ANC service at least once, for their recent pregnancy. Among mothers who attained ANC for their last pregnancy, 42 (6.2%) were attained 4 and more times, whereas, 135 (19.5%) and 172 (28%) were attained 2–3 and one times respectively. Three hundred fifty (73.1%) of study participants delivered their recent child at a health facility (i.e. Health center or hospital), whereas their counterparts delivered outside the health facilities. However, only 179 (37.4%) of them had visited health facilities for postnatal care.

### Knowledge of PCC among reproductive age group women

Among the total of 669 participants, only 148 (22.1%) of women have heard about PCC before and the rest majority of them 521 (77.9%) didn’t heared about PCC. For those who have heard about PCC; the major source of information was health workers 54 (8.1%). Fifty two (7.8%), 28 (4.2%) and 14 (2.1%) of them have heard from the mass media, school and family/relatives respectively. The minimum and maximum knowledge score of the participants was 1 and 20 respectively. More than half of the study participants 490 (73.2%) had inadequate knowledge of PCC, the rest 179 (26.8%) of the study participants had good PCC knowledge.

**Fig. 1:**
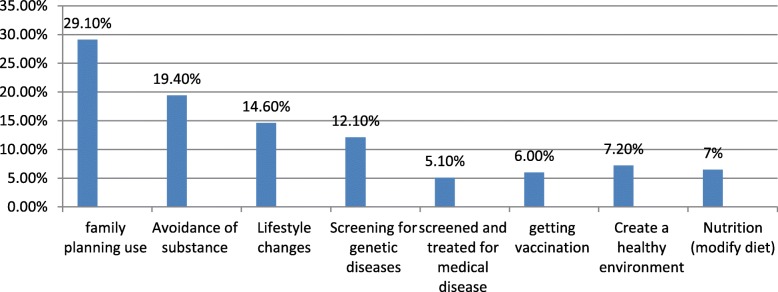
Women’s knowledge of the preconception care component before getting pregnant in the West Shewa zone, Oromia, regional state, 2018

During the household interview, the study participants were asked what should be done before conception (components of PCC). Family planning was mentioned profusely than the rest of PCC components 195 (29.1%). Avoidance of substance 130 (19.4%), getting vaccination 40 (6%) and screened and treated for disease 34 (5.1%) for getting pregnant were components of PCC mentioned by the study participants (Fig. 1).

Regarding women’s knowledge on untreated health problem that could affect the fetus; STIs including HIV/AIDS 481 (71.9%), Diabetes mellitus 331 (49.5%), Obesity 167 (25%), Epilepsy208 (31.1%) and alcohol consumption174 (26.0%) are most frequently mentioned as untreated health problem which can affect the fetus, whereas cigarette smoking 112 (16.7%), genetic problem126 (18.8%) and exposure to environmental hazard 83 (12.4%) were mentioned by few (Table 2**).**

**Table 2:** Women’s knowledge of untreated health problem, social and cultural behaviors affect the fetus and pregnancy outcome in the West Shewa zone, Oromia, regional state, 2018

Variables		Frequency	Percentages
Diabetes mellitus	Yes	331	49.5
	No	338	50.5
Epilepsy	Yes	208	31.1
	No	461	68.9
Obesity	Yes	167	25.0
	No	502	75.0
STIs and HIV/AIDS	Yes	481	71.9
	No	188	28.1
Heart disease, including hypertension	Yes	258	38.6
	No	411	61.4
Stress and depression	Yes	112	16.7
	No	557	83.3
Genetic problem	Yes	126	18.8
	No	543	81.2
Illegal drugs intake	Yes	15	2.2
	No	654	97.8
Cigarette smoking	Yes	112	16.7
	No	557	83.3
Alcohol consumption	Yes	174	26.0
	No	495	74.0
Exposure to environmental hazard	Yes	83	12.4
	No	586	87.6

### Uptakeof preconception care

Only Ninety-seven (14.5%) women have utilized PCC services, and the rest majority of the 572 (85.5%) have not utilized PCC. The study participants were asked the uptake level of PCC services and the most utilized preconception service were family planning 251 (37.5%), stop taking of illegal drugs 183 (27.3%), taking immunization against tetanus 145 (21.65%) and received preconception screening for medical and genetic conditions 118 (17.6%). The least component mentioned by participants was a cessation of alcohol and cigarettes smoking (11.6%), consumption of folic acid supplementation before pregnancy 52 (7.7%). 150 (22.4%) study participants also weight monitored before conception.

### Bivariate and multivariate logistic regression analysis of knowledge and uptake of PCC among reproductive age group

The study revealed that, five factors found to show significantassociation with the knowledge of PCC. A woman who had a better educational status were three to four times more likely to have good knowledge than women who had lower educational status. A reproductive age group woman who has regular employment is two times more likely to have good knowledge than students and housewives. Women who have a history of institutional delivery are two times more likely to have good knowledge of PCC than those women who don’t have a history of institutional delivery similarly women who utilize PNC and had a history of using modern contraceptive are five times and two times more likely have good knowledge compared to their reference group.

Women who had better family incomes greater than 2800 ETB per month are four times more likely to utilize PCC. Women who utilize PNC service nearly six times more likely to utilize PCC than those who don’t utilize PNC. Having good knowledge of PCC has shown a positive association with the uptake of PCC. A woman who was a good knowledge of PCC four times more likely to utilize PCC than women who have poor knowledge of PCC(Table 3**)**.

**Table 3:** Bivariate and multivariate logistic regression analysis of knowledge and uptake of PCC among reproductive age group in the West Shewa zone, Oromia, regional state, 2018

variables	Knowledge of PCC		COR (95%CI)	AOR (95%CI)
	Good	Poor		
Occupation				
House wife	50 (7.5%)	199 (29.7%)	1.00	1.00
Student	9 (1.3%)	17 (2.5%)	2.10(.88–5.00)	2.15(.88–5.23)
Gov’t employee	48 (7.2%)	87 (13%)	2.19 (1.37–3.51)	1.80 (1.01–3.22)
NGO employee	35 (7.5%)	57 (8.5%)	2.44 (1.45–4.12)	2.11 (1.20–3.71)**
Private business	37 (5.5%)	130 (73.2%)	1.133(.70–1.82)	1.01(.61–1.65)
Educational status of women				
No formal school	12 (1.8%)	72 (10.8%)	1.00	
1–4 grade completed	13 (1.9%)	79 (11.8%)	.99 (0.42–2.30)	1.165(.46–2.96)
5–8 grade completed	61 (9.1%)	147 (22%)	2.49 (1.23–4.915)	2.82 (1.91–8.81)
9–12 completed	53 (7.9%)	116 (17.3%)	2.74 (1.37–5.47)	3.28 (1.51–7.13)**
College and above	40 (6.0%)	76 (11.4%)	3.16 (1.53–6.49)	4.12 (1.22–6.52)**
Have you ever delivered baby in health institution				
Yes	134 (20.1%)	310 (46.4%)	1.73 (1.83–3.78)	1.21 (1.31–7.33)**
No	45 (6.6%)	180 (26.9%)	1.00	1.00
utilize PNC service				
Yes	96 (14.3%)	83 (12.3%)	5.67 (3.89–8.26)	5.02 (3.22–7.84)**
No	83 (12.3%)	407 (60.9%)	1.00	1.00
modern family planning use				
Yes	106 (15.8%)	230 (34.4%)	1.64 (1.08–4.22)	1.44 (1.37–6.98)**
No	73 (10.9%)	260 (38.9%)	1.00	
Factors associated with uptake of PCC				
variables	**Uptake of PCC**		**COR (95%CI)**	**AOR (95%CI)**
	**Yes**	**No**		
Monthly income				
< = 1500	31 (4.6%)	241 (36%)	1.00	1.00
1501–2000	23 (3.4%)	159 (23.8%)	1.12(.63–1.99)	.695(.43–1.12
2001–2800	11 (1.6%)	40 (6%)	2.13(.99–4.59)	.74(.43–1.25
2801+	32 (4.8%)	132 (19.7%)	**1.88 (1.10–3.22)**	4.1 (1.57–9.35)*
utilize PNC service				
Yes	61 (9.1%)	118 (17.6%)	**6.33 (3.94–10.17)**	6.33 (3.94–10.17)*
No	36 (5.4%)	454 (67.9%)	**1.00**	**1.00**
Knowledge about PCC				
Poor Knowledge	41 (42.3%)	449 (78.5%)	**1**	**1**
Good Knowledge	56 (57.7%)	123 (21.5%)	**4.99(3.20–7.82)**	4.3(2.67–6.98)*

* *P*-value< 0.05 statically significant, **P-value< 0.01

## Discussion

The study revealed that knowledge of PCC by reproductive age group women was 179 (26.8%), this finding is higher than studies conducted in Sudan (11.1%), Iran (14%) and Nepal (15.6%) [14–16]. However, it is lower than the findings from Saudi Arabia (57.2%), Jordan (85%), and in the USA among low-income Mexican American groups (76%) [17–19]. The low knowledge level in this study might be due to the relatively low media coverage in Ethiopia concerning PCC, which showed there is a need to broaden media coverage in the country.

Women who learned up to 9–12 grade of education are 3.28 times and those who learned college and above 4.12 times were more likely to have better knowledge on PCC than women who had lower educational status. A study from Iran, Nigeria, Sri Lanka, and Gojjam, also in line with this study [10, 18, 20, 21]. This might be due to the might be due to educated women can discuss more sensitive issues openly and freely since they become closer and familiarized with each other. Besides, women with some basic level of education had better understand the complications associated with not to use PCC.

This study also indicated that having a history of family planning use is significantly associated with knowledge of PCC. Those mothers who use family planning more than 1 year 1.44 times more likely to have good knowledge about PCC when compared to those who didn’t utilize it. This is supported by the studies conducted in France, Sudan and Gojjam [10, 16, 22]. This might be due to women who get pregnancy counseling, including PCC, which is being given in the family planning unit; women who used family planning might have information regarding PCC.

The occupational status of women was also significantly associated with knowledge of PCC in this study. Reproductive age group women who have regular employment are 2.11 times more likely to have good knowledge than students and housewives. But Study from Srilanka [23] contrasts with this study, showing that no significant association between occupation and w omen’s knowledge about PCC. This might be due to the socio-demographic difference of the study participants.

Regarding the prevalence of uptake PCC, about 97 (14.5%) of women of reproductive age group have utilized of PCC. This is similar to a study conducted in Ethiopia 13.4%, Nigeria 10.5% and a study conducted in France (15.8%) [11, 13, 24].

In this study, mothers who get monthly income / total family with monthly income 2801+ ETB were 4.1 times more likely to uptake PCC compared with those who can get ≤1500 ETB. This might be due to those mothers in low socio-economic status cannot afford their health expense.

In this study knowledge of PCC is significantly associated with uptake of PCC. A woman who was a good knowledge of PCC 4.3 times more likely to utilize PCC than women who have poor knowledge of PCC. This is comparable with the study conducted in France [25].

### Limitations of the study

limitation of this study is that it is purely quantitative and doesn’t have the capacity to explore the myriad of contextual and social factors that may be limiting women in PCC service, so it would be very worthwhile to suggest future qualitative research to follow-up on these findings.

## Conclusion

This study found that only one-quarter of the women in the study have good knowledge of PCC and uptake of PCC among the study participant is found to be very low. History of institutional delivery, PNC service utilization, and history of using modern contraceptives, educational status and occupation are factors that are significantly associated with good knowledge of PCC. On the other hand factors like family monthly income, history of postnatal care service and good knowledge of PCC had shown significant association towards the uptake of PCC. Therefore, establishing PCC strategies which can address all the components of PCC and integration of services with other maternal and child health service will be essential when designing effective implementation strategies for improving delivery and uptake of PCC and advocating women’s education and family planning use are important.

## Supplementary information

**Additional file 1.** Information sheet and Informed consent statements.

## Data Availability

Full data for this research is available through the corresponding author upon request.

## Abbreviations

AOR: Adjusted Odds Ratio
CI: Confidence interval
COR: Crude Odds Ratio
EDHS: Ethiopian Demographic Health Survey
PCC: Preconception Care
SRS: Simple Random Sampling
WHO: World Health Organization
