# Supplementary material for: Knowledge, uptake of preconception care and associated factors among reproductive age group women in West Shewa zone, Ethiopia, 2018
Source: BMC Womens Health. 2020 Feb 19;20:30. doi: 10.1186/s12905-020-00900-2 (PMC7029592; doi:10.1186/s12905-020-00900-2)
Supplement: Supplementary file 1 — Additional file 1. Information sheet and Informed consent statements. [file 12905_2020_900_MOESM1_ESM.docx]

**Appendix I. Information sheet and Informed consent statements**

**Information sheet**

Introduction: Hello! Sir/Madam my name is ___________and I came from_________________. We are conducting a study on ‘knowledge ,uptake of PCC and associated factors among reproductive age group women in Oromia regional state, west shoa zone. The aim of this study is to assess the knowledge and uptake of PCC. The results of this study will be helpful overcome barriers towards preconception care uptake of PCC so that recommendations will be made to responsible organizations to fill those gaps.

**Consent statements(to be read by data collector for the study participants)**

I am going to ask you questions about knowledge, uptake of PCC and associated factors. You are randomly selected to participate in the study. Your participation in this study doesn’t involve any direct risk or benefit for you, but is very useful to improve the maternal and neonatal health in this area. Your name will not be appearing on this questionnaire, and all the information you provide me will be strictly confidential. It takes 30 minutes to finish the interview and you are not obliged to answer any question you don’t wish to answer, and you can also end this interview at any time, if you wish to do so. Would you like to participate in the study?

Yes________ No_________

Interviewer, if the answer is “Yes” please let the participant to sign (i.e. Either using their signature or fingerprint) below to certify her verbal consent to take part voluntarily in the study. Otherwise, thank the client, conclude the conversation and file the questionnaire.

Signature_________

Date _____________ Identification No. __________

Contact address of principal investigator: - mobile: +251917675752

email:danibelema@gmail.com

**Part I: Socio -demographic factors**

| **Sr.No** | **Questions** | **Responses** |
| --- | --- | --- |
| 1 | What is your age? | In years................. |
| 2 | What is your marital status? | 1. Married 2. Divorced 3. Widowed  4. Cohabited 5. Other, specify....... |
| 3 | What is your religion? | 1. Orthodox 2. Muslim  3. Protestant 4. Catholic 5. Other, specify....... |
| 4 | What is your ethnicity? | 1. Amhara 2. Oromo  3. Tigray 4. Others, specify---------- |
| 5 | What is your occupation? | 1 . Housewife 2. Student  3. Government employee 4. NGO employee  5 Private business 6. Other, specify....... |
| 6 | What is your educational status? | 1. No formal school 2. 1- 4 grade completed  3. 5-8 grade completed 4. 9-12 completed  5. College and above |
| 7 | Monthly income | In number ___________ |

**Part II: Questions related to past obstetric history**

| 8 | How many deliveries have you ever had (parity)? | In number………………….. |
| --- | --- | --- |
| 9 | Number of live children? | In number............ |
| 10 | Have you ever utilized the ANC service for any of your previous pregnancy? | 1 Yes 2. No |
| 11 | If the answer is yes to QNO 10, for how many times you attend? | In number_______ |
| 12 | Have ever gave birth baby in health institution? | 1 Yes 2. No |
| 13 | If the answer is yes toQ12 where did you gave birth your recent child? | 1. Health centers 2. Hospital 3. Others |
| 14 | Have you ever utilized PNC service for your recent child? | 1 Yes 2. No |
| 15 | Have you ever used family planning before? | 1 Yes 2. No |

**Part III: Questions related to knowledge of preconception care**

| 16 | Have you ever heard about preconception care? | 1 Yes 2. No |
| --- | --- | --- |
| 17 | If yes, for Q16 where did you heard?  (Multiple answers are possible) | 1. Family/relatives 2. Health institutions  3. Mass media 4. School 5. Other, Specify.......... |
| 18 | Which Parents’ untreated health problem affects the fetus?  Multiple answers are possible | Yes No  A. Diabetes mellitus 1 2  B. Epilepsy 1 2  C. Obesity 1 2  D. STI, including HIV 1 2  E. Heart disease, including hypertension 1 2  F. Stress and depression 1 2  G. Genetic problem 1 2 |
| 19 | What parents’ social and cultural behaviors affect the pregnancy outcome?  Multiple answers are possible | Yes No  A. Cigarette smoking 1 2  B. Alcohol consumption 1 2  C. Exposure to environmental hazardous 1 2  D. Illegal drugs intake 1 2 |
| 20 | Do you know that a woman should be on a family planning method during the preconception period? | 1 Yes 2. No |
| 21 | Do you know that a woman should be vaccinated against tetanus and rubella before she conceives? | 1 Yes 2. No |
| 22 | Do you know that a woman should be screened for medical conditions, e.g., blood pressure, anemia, and diabetes and HIV e.t.c? | 1 Yes 2. No |
| 23 | Do you know that a woman should stop using alcohol, smoking cigarette and illicit drugs before conception? | 1 Yes 2. No |
| 24 | Do you know that a woman should undergo weight monitoring and use folic acid before conception? | 1 Yes 2. No |
| 25 | Do you know that a woman should be screened for familial diseases such sickle cell anemia before conception? | 1 Yes 2. No |
| 26 | Do you know a woman should create healthy environments (free from radiation, chemical and stress) before pregnancy? | 1 Yes 2. No |

**Part III: Questions related to uptake of preconception care**

| 1 | Did you visit health institutions before pregnancy? | 1 Yes 2. No |
| --- | --- | --- |
| 2 | If yes to question number 2, why did you visit health institutions before pregnancy?  Multiple answers are possible | Yes No  A. To take folic acid 1 2  B. To screened and treated for disease 1 2  C. To get Vaccination 1 2  D. To receive medical advice 1 2  E. To use Family planning 1 2 |
| 3 | Did you maintain/adjust your weight before pregnancy for the sake of your current pregnancy? | 1 .Yes 2. No |
| 4 | If yes, for question number 3, how did you maintain weight?  Multiple answers are possible | Yes No  A. Modify diet 1 2  B. Exercise 1 2 |
| 5 | Did you avoid taking the substance before pregnancy? | 1 .Yes 2. No |
| 6 | If yes, for question number 5, what substances did you avoid?  Multiple answers are possible | Yes No  A. Cigarette smoking 1 2  B. Alcohol consumption 1 2  C. Illegal drugs 1 2 |
| 7 | Did you create healthy environments before pregnancy? | 1. Yes 2. No |
| 8 | If yes, for question number 7, how did you create a healthy environment?  Multiple answers are possible | Yes No  A. Free from environmental Radiation 1 2  B. Free from environmental Chemicals 1 2  C. Free from stressors 1 2 |

Thank you for your cooperation!!!!
